# Beyond Frequency: Generative AI Engagement Quality, Psychological Well-Being, and Digital Inequality Among Chinese University Students

**DOI:** 10.3390/bs16101942

**Published:** 2026-10-19

**Authors:** Zeyuan Cao, Yue Zhai, Rong Zheng

**Affiliations:** 1 Department of Social Work, School of Social Development, East China Normal University, Shanghai 200241, China; caozeyuan0410@163.com; 2 School of Humanities and Social Sciences, Guangxi Medical University, Wuming Campus, No. 345 Wuqiang Road, Wuming District, Nanning 530100, China; zhaiyue5344@163.com

**Keywords:** psychological well-being, AI literacy, resilience, digital inequality, university students

## Abstract

Generative artificial intelligence (GenAI) is now embedded in university students’ academic and everyday lives, yet frequency-based measures provide limited insight into why similar levels of use are associated with different psychological well-being outcomes. Using cross-sectional survey data from 3283 university students enrolled predominantly at institutions in Guangxi, China, this study examined GenAI engagement at three levels: overall use frequency, specific use purposes, and engagement quality reflected in self-perceived AI competence, self-reported critical AI literacy, and AI unavailability anxiety. Covariate-adjusted regressions, parallel statistical indirect-path models with 5000 bootstrap resamples, moderation analyses, and family-resource models were estimated. Overall use frequency was not associated with depressive symptoms or life satisfaction in baseline models. By contrast, use purposes showed heterogeneous associations, and engagement-quality indicators were more consistently related to well-being. More frequent use was associated with greater perceived competence and literacy but also with greater unavailability anxiety. Favorable indirect associations through competence and literacy partly offset the adverse association through anxiety, although the total indirect association with depressive symptoms was not statistically significant. The inverse association between self-perceived AI competence and depressive symptoms was statistically significant at low, but not average or high, levels of psychological capital, while family economic, cultural, and network resources showed distinct associations with competence and literacy. These findings direct attention beyond GenAI exposure frequency to the quality of engagement and the institutional conditions supporting equitable access to critical AI literacy.

## 1. Introduction

Generative artificial intelligence (GenAI) is becoming embedded in university learning and everyday academic work. Students use conversational AI to search for information, generate explanations, translate materials, draft text and code, summarize documents, and solve academic problems. This expansion has intensified debate over whether GenAI should be understood primarily as a learning resource, a source of risk, or both. Reviews of ChatGPT-3.5 and related systems point to opportunities for feedback and productivity while also noting concerns about inaccurate outputs, academic integrity, and overreliance (Lo, 2023). Tlili et al. (2023) similarly argue that the educational value of GenAI depends on how students engage with it rather than on access alone. UNESCO (2023) consequently places human agency, critical judgment, and responsible use at the center of educational guidance on GenAI. Empirical evidence nevertheless remains mixed. Abbas et al. (2024), for example, report adverse academic and social consequences associated with problematic ChatGPT use. These contrasting findings suggest that the implications of GenAI cannot be understood adequately by asking only whether, or how often, students use it.

A central problem is that exposure is not equivalent to experience. Research on digital well-being has shown that broad indicators such as screen time, duration, or frequency can obscure substantial variation in what users actually do. Average associations between digital technology use and well-being are often modest when engagement is represented only by overall exposure (Orben & Przybylski, 2019). The differential-susceptibility perspective likewise emphasizes that media effects vary across individuals and contexts rather than operating uniformly (Valkenburg & Peter, 2013). Vanden Abeele (2021) conceptualizes digital well-being as dynamic and situational, while Valkenburg (2022) argues that consequences depend on the form and context of use. GenAI makes this distinction especially important because the same interface can support very different activities. Using AI to clarify a concept or translate a passage is not equivalent to routinely delegating writing, programming, or problem solving, even when overall frequency is similar. Unlike conventional screen-time measures, GenAI engagement involves interactive prompting, contingent feedback, and opportunities to delegate or verify cognitive tasks, making its educational and psychological associations especially dependent on how the technology is used.

Engagement quality also depends on what students are able to do with AI and how they relate to it. AI literacy involves more than technical familiarity; it includes understanding AI, evaluating its outputs, and recognizing ethical and practical limitations (Long & Magerko, 2020; Ng et al., 2021). University students differ substantially in these capacities despite widespread access to GenAI (Hornberger et al., 2025). Competence and critical literacy may help students retain control over AI-supported tasks, whereas reliance on uninterrupted access may introduce a different vulnerability. More frequent use may therefore be accompanied by both resource accumulation and reliance-related risk rather than a single uniformly beneficial or harmful process.

Three questions follow from this distinction. First, it remains unclear whether overall GenAI use frequency is associated with psychological well-being once specific purposes of use are considered. Second, little is known about whether frequent use is simultaneously associated with potentially protective resources, such as AI competence and literacy, and potentially adverse responses, such as anxiety when AI is unavailable. Third, opportunities to develop higher-quality engagement may be socially patterned because students enter university with different psychological and family resources.

Using survey data from Chinese university students, the present study distinguishes overall use frequency from eight specific use purposes and examines three dimensions of engagement quality: self-perceived AI competence, critical AI literacy, and AI unavailability anxiety. It tests competing statistical pathways linking use frequency with depressive symptoms and life satisfaction, examines psychological capital as a boundary condition of the competence–well-being association, and considers whether family economic, cultural, and network resources are differentially associated with competence and literacy. By separating exposure from engagement quality and placing both within psychological and social contexts, the study seeks to explain why similar levels of use may correspond with different psychological well-being.

## 2. Literature Review

### 2.1. From Use Frequency to Engagement Quality

A growing body of literature in higher education distinguishes simple access to GenAI from the quality and purpose of engagement. Farrokhnia et al. (2024), for example, show that ChatGPT can support adaptive learning and efficient knowledge work while also creating risks related to accuracy, integrity, and dependency. Evidence from Chinese higher education likewise suggests that GenAI use may be associated with both academic performance and psychological well-being, but these associations vary with the way use is conceptualized (Shahzad et al., 2025). A frequency measure cannot capture these functional differences. Daily information search and daily writing substitution may represent very different forms of engagement even when they produce the same frequency score. We therefore first examine whether overall GenAI use frequency is associated with depressive symptoms and life satisfaction, and then assess whether specific use purposes show distinct associations after overall frequency is taken into account.

### 2.2. AI Competence, AI Literacy, and Unavailability Anxiety

The quality of GenAI engagement can be understood partly through the capabilities students develop and the forms of reliance that accompany use. Self-perceived AI competence refers here to students’ perceived ability to use AI effectively, including formulating appropriate instructions and evaluating output quality. The self-reported AI literacy index is broader, encompassing conceptual knowledge, critical evaluation, and ethical awareness. Recent measurement studies translate this multidimensional view into scales that distinguish technical familiarity from evaluative and ethical judgment (Carolus et al., 2023/2026; Laupichler et al., 2023), while short-form validation shows that these dimensions can be assessed parsimoniously without reducing literacy to simple tool use (Koch et al., 2024). Cross-national evidence also indicates substantial variation in AI literacy among university students (O’Dea et al., 2026; Wu et al., 2025).

Frequent interaction with GenAI may provide opportunities to practice prompting, compare outputs, and learn the boundaries of automated responses. More frequent use may therefore be associated with greater perceived competence and literacy. Yet repeated use can also increase the perceived importance of continued access. Students may experience apprehension about AI even while recognizing its usefulness (Cengiz & Peker, 2025), and technology-acceptance research identifies anxiety and self-efficacy as distinct responses to AI-supported learning (Chen et al., 2024). Other studies link academic stress with problematic AI use (S. Zhang et al., 2024), while work on cognitive offloading raises concerns that habitual delegation may weaken users’ sense of cognitive ownership (Su et al., 2026). We conceptualize AI unavailability anxiety as self-reported discomfort related to interruptions in AI access, distinct from trait-like technological dependence.

These dimensions may have different implications for well-being. Competence may support a sense of control in demanding tasks, while critical literacy may help students calibrate trust, detect errors, and decide when independent verification is needed. Recent evidence links AI literacy and self-regulated learning with writing performance and student well-being (Shi et al., 2025). By contrast, unavailability anxiety may signal fewer perceived alternatives for coping with uncertainty or completing tasks. The coexistence of these processes implies competing statistical pathways: frequent use may be favorably associated with well-being through competence and literacy, yet adversely associated through stronger unavailability anxiety.

### 2.3. Psychological Capital and Digital Inequality

The relevance of AI competence may also depend on the psychological resources students already possess. Psychological capital brings together self-efficacy, hope, resilience, and optimism as resources that support adaptive functioning (Luthans et al., 2007). A recent review of Chinese university students similarly links psychological capital with better mental-health outcomes (Z. Zhang, 2026). Resource-substitution theory provides a useful way to think about the interaction between these resources and digital competence. Ross and Mirowsky (2006) argue that one resource may become especially consequential when another is scarce. Applied to GenAI, perceived AI competence may provide additional control for students who have fewer general psychological resources for managing uncertainty and academic demands. This logic leads to the expectation that the inverse association between AI competence and depressive symptoms will be stronger among students with lower psychological capital. Individual psychological resources may condition the competence–well-being association, whereas family resources may shape opportunities to acquire and evaluate GenAI-related capabilities.

Opportunities to develop AI competence and literacy may also be socially patterned. The digital-divide literature has moved from differences in access toward inequalities in skills and in the benefits obtained from digital technologies. Hargittai (2002) described these skill differences as a second-level digital divide, while van Deursen and Helsper (2015) extended the framework to inequalities in outcomes. Helsper (2021) further argues that digital inequalities are embedded in broader distributions of economic, cultural, and social resources. This perspective is increasingly relevant to GenAI. Economic resources can support devices, paid services, and repeated opportunities for practice; cultural resources may foster evaluative habits and information literacy; and network resources can provide advice from more experienced users. Recent studies show that GenAI performance and perceived benefits remain associated with students’ social and cultural capital even where access to the technology is widespread (Tang, 2025). Evidence from U.S. college students likewise indicates that educational benefits from ChatGPT are unevenly distributed across student groups (C. Zhang et al., 2025).

### 2.4. The Present Study

**Figure 1:**
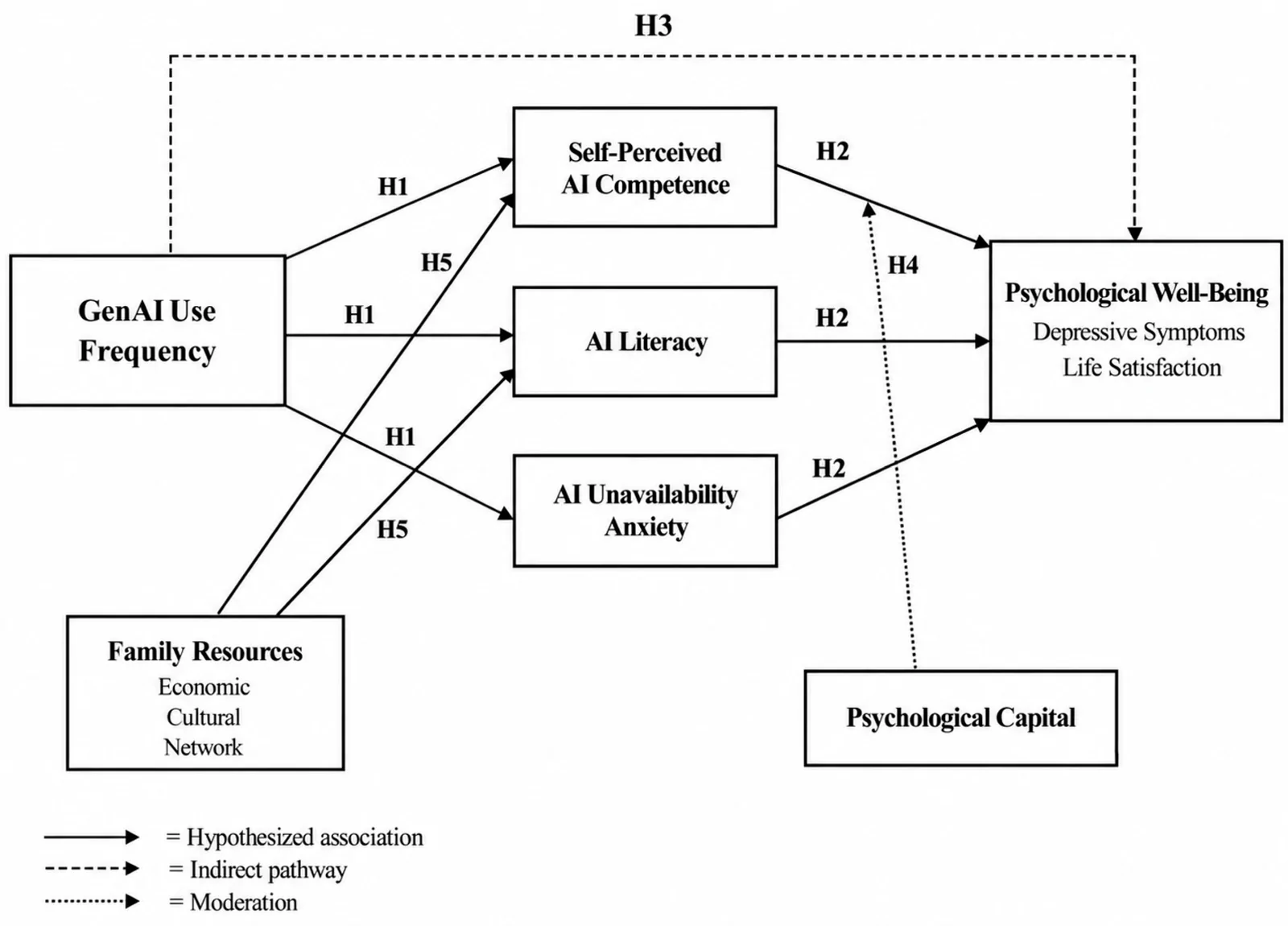
Conceptual framework.

Against this background, this study examines GenAI engagement among Chinese university students through a model that distinguishes exposure from engagement quality and places both within students’ psychological and family-resource contexts. The analysis first compares overall use frequency with eight specific use purposes. It then estimates parallel pathways through AI competence, AI literacy, and AI unavailability anxiety; examines psychological capital as a boundary condition of the competence pathway; and considers family economic, cultural, and network resources as antecedents of competence and literacy. This organization moves beyond the question of how often students use GenAI to examine how they use it, what capacities and forms of reliance accompany that use, and how opportunities for higher-quality engagement are socially patterned. The conceptual framework guiding this study is presented in Figure 1. Accordingly, the following hypotheses were proposed:

**Hypothesis** **1.** *More frequent GenAI use is positively associated with self-perceived AI competence, AI literacy, and AI unavailability anxiety.*

**Hypothesis** **2.** *Greater self-perceived AI competence and self-reported AI literacy are associated with fewer depressive symptoms and greater life satisfaction, whereas greater AI unavailability anxiety is associated with more depressive symptoms and lower life satisfaction.*

**Hypothesis** **3.** *GenAI use frequency has favorable statistical indirect associations with psychological well-being through self-perceived AI competence and AI literacy and an adverse statistical indirect association through AI unavailability anxiety.*

**Hypothesis** **4.** *The inverse association between self-perceived AI competence and depressive symptoms, and the corresponding indirect association through competence, are stronger among students with lower psychological capital.*

**Hypothesis** **5.** *Family economic resources are positively associated with self-perceived AI competence, cultural resources with AI literacy, and network resources with both competence and literacy.*

## 3. Method

### 3.1. Data and Sample

This study used data from the University Students’ Comprehensive Health Development and Career Planning Survey, which was administered to students enrolled at multiple higher education institutions in Guangxi, China. The questionnaire covered demographic characteristics and family background, physical and psychological health, career development, everyday digital behaviors, and the use of generative artificial intelligence (GenAI). A total of 3456 valid questionnaires were collected. After checking for duplicate responses and excluding cases with substantial missing data, 3283 participants were retained in the final analytical sample.

### 3.2. Measurement

#### 3.2.1. GenAI Use Frequency and Use Purposes

GenAI use frequency was measured with one item asking how often students used AI tools for study or work. Examples included ChatGPT, DeepSeek, Kimi, Doubao, iFlytek Spark, and Wenxin Yiyan. Response categories ranged from never to almost every day and were reverse-coded so that higher values represented more frequent use (1 = never; 5 = almost every day). Students also selected all applicable purposes from eight binary options: information search and knowledge questions, translation, writing assistance, programming and debugging, data organization and analysis, creative generation, summarization of long documents or videos, and chat or emotional companionship.

#### 3.2.2. AI Competence

Self-perceived AI competence was assessed with a single self-rating of the ability to use AI tools effectively, including formulating precise instructions and evaluating output quality. Responses were reverse-coded so that scores ranged from 1 (completely unable) to 5 (very proficient), with higher scores indicating greater perceived competence.

#### 3.2.3. AI Literacy

AI literacy was measured with three items assessing understanding of basic AI concepts, the ability to critically evaluate the accuracy and reliability of AI-generated content, and awareness of ethical risks such as privacy leakage, algorithmic bias, and academic misconduct. Responses were recorded on five-point agreement scales and averaged, with higher scores indicating greater literacy. The item content follows conceptual work that treats AI literacy as involving knowledge, critical evaluation, and ethical judgment rather than mere familiarity with AI tools (Long & Magerko, 2020; Ng et al., 2021). It is also consistent with recent scale-development work emphasizing metacognitive and evaluative competencies (Carolus et al., 2023/2026; Laupichler et al., 2023). The three-item index showed good internal consistency in the present sample (Cronbach’s α = 0.826).

#### 3.2.4. AI Unavailability Anxiety

AI unavailability anxiety was measured by the single item, “When I cannot use AI, I feel anxious,” rated from 1 (strongly disagree) to 5 (strongly agree). Higher scores indicate greater self-reported anxiety associated with interruptions in AI access.

#### 3.2.5. Depressive Symptoms and Life Satisfaction

Depressive symptoms were assessed with 10 items corresponding to the short form of the Center for Epidemiologic Studies Depression Scale (CES-D-10). The original CES-D was developed to measure depressive symptomatology in general populations (Radloff, 1977), and the 10-item short form has demonstrated adequate screening performance (Andresen et al., 1994). The survey items referred to the previous week and covered depressed mood, difficulty concentrating, effort, fear, sleep problems, loneliness, and positive affect. Positive items were reverse-scored before summation; higher values indicate more depressive symptoms. Internal consistency was α = 0.838. No clinical cutoff was applied because the analysis treated the score as a continuous symptom measure. Life satisfaction was assessed with a single five-point item asking respondents how satisfied they were with their lives overall. Single-item global life-satisfaction measures have shown acceptable validity in large population samples (Cheung & Lucas, 2014).

#### 3.2.6. Psychological Capital

Psychological capital was assessed using the established 26-item Chinese Positive Psychological Capital Questionnaire (PPQ; K. Zhang et al., 2010). This culturally developed measure comprises self-efficacy (7 items), resilience (7 items), hope (6 items), and optimism (6 items). Items 8, 10, 12, 14, and 25 were reverse-coded before calculating the mean total score. Cronbach’s α was 0.927 for the full questionnaire and 0.898, 0.719, 0.838, and 0.890 for the four dimensions, respectively (Table S2). An ordinal CFA of the 26 five-category items using polychoric correlations and diagonally weighted least squares (DWLS) supported both the correlated four-factor model with an orthogonal wording-method factor (CFI = 0.994, TLI = 0.994, RMSEA = 0.057, RMSR = 0.050) and its second-order counterpart (CFI = 0.992, TLI = 0.991, RMSEA = 0.066, RMSR = 0.056; Table S1). Together with the full-scale reliability (α = 0.927), these results support using the overall PPQ score.

#### 3.2.7. Family Resources and Covariates

Primary regression models adjusted for gender, hukou registration, annual family income, material assets before age 16, father’s education, mother’s education, books in the home before age 16, frequency of cultural activities with parents before age 16, parental ability to mobilize social ties for educational support, grade fixed effects, and institution fixed effects. For the inequality analyses, standardized resource composites were created to distinguish economic, cultural, and network resources. Economic resources averaged standardized family income and material assets and were then re-standardized. Cultural resources were constructed analogously using father’s education, mother’s education, books in the home, and parent–child cultural activities. Network resources standardized parental ability to mobilize social relationships. The three standardized composites were entered simultaneously so that their coefficients captured distinct resource dimensions rather than undifferentiated socioeconomic status.

### 3.3. Analytical Plan

The analysis proceeded in five stages. First, descriptive statistics summarized the focal variables and GenAI use purposes. Second, ordinary least squares models with fully standardized coefficients (β) were estimated for depressive symptoms and life satisfaction. Model 1 included GenAI use frequency, Model 2 added eight use-purpose indicators, and Model 3 further included self-perceived AI competence, self-reported AI literacy, and AI unavailability anxiety. All models adjusted for the specified covariates and used HC3 robust standard errors (J. G. MacKinnon & White, 1985). Standard errors for standardized coefficients were scaled consistently.

Third, parallel-path models estimated indirect associations through self-perceived AI competence, self-reported AI literacy, and AI unavailability anxiety, with 95% confidence intervals based on 5000 nonparametric bootstrap resamples (D. P. MacKinnon et al., 2007). These indirect associations and their bootstrap confidence intervals were reported on a fully standardized scale. A residual-correlation sensitivity analysis examined hypothetical unmeasured mediator–outcome confounding in the three-mediator linear specification, with 5000 bootstrap resamples and the other two mediator–outcome residual correlations fixed at zero when varying one pathway (Table S6). This procedure probes specified departures from the no-unmeasured-mediator–outcome-confounding assumption; it does not identify causal mediation. The parallel-path equations, estimated separately for depressive symptoms and life satisfaction, are:

$$M_{\mathrm{ki}}=\alpha_{0k}+a_{k}F_{i}+\delta_{k}^{⊤}C_{i}+u_{\mathrm{ki}},\;\;k=1,2,3$$

$$Y_{i}=\beta_{0}+c^{\prime }F_{i}+\sum_{k=1}^{3}b_{k}M_{\mathrm{ki}}+\gamma^{⊤}C_{i}+\varepsilon_{i}$$

Here F is GenAI use frequency; M1, M2, and M3 denote self-perceived AI competence, self-reported AI literacy, and AI unavailability anxiety, respectively; C contains the covariates. Residuals in the three mediator equations may correlate. Each statistical indirect association is the product of its two path coefficients. The three-mediator equations describe the parallel indirect-path analysis; psychological-capital moderation is examined separately for the competence-specific path, with its conditional-association formula and standardized Hayes index specified in Supplementary Methods S1.3.

Fourth, moderation analyses tested whether psychological capital conditioned the association between self-perceived AI competence and depressive symptoms. Simple slopes were estimated at −1 SD, the mean, and +1 SD, and moderated mediation was assessed using Hayes’ index with 5000 bootstrap resamples (Hayes, 2015). Finally, economic, cultural, and network resources were modeled as predictors of GenAI engagement indicators, with Benjamini–Hochberg correction for multiple testing (Benjamini & Hochberg, 1995). Sensitivity analyses included academic-major fixed effects, first-year-only models, and alternative measures of AI literacy and unavailability anxiety. To characterize digital inequality, lower and upper resource quartiles and agricultural versus non-agricultural hukou groups were compared descriptively (Tables S3 and S4); adjusted hukou-by-resource interactions were then estimated with the three resources included jointly (Table S5). For the competence–depressive-symptom association moderated by psychological capital, the moderated-mediation index is:

$${\mathrm{Index}}_{\mathrm{MM}}=a_{1}b_{1,\mathrm{PC}}$$

Here the interaction coefficient denotes the self-perceived AI competence × psychological-capital term in the depressive-symptom model. Further equations, standardization details, and sensitivity calculations appear in Supplementary Methods S1. The competence-specific expression should not be interpreted as a moderated total indirect association across all three intermediates.

## 4. Results

### 4.1. Descriptive Characteristics of GenAI Engagement

Table 1 presents descriptive statistics for the focal variables. GenAI use was common in the sample, with a mean frequency of 4.27 on the five-point scale. Mean self-perceived AI competence was 3.55 and mean AI literacy was 3.33. AI unavailability anxiety averaged 2.85, indicating meaningful variation rather than uniformly high distress. As shown in Table 2, information search was the most frequently reported use purpose (87.0%), followed by writing assistance (65.6%) and translation (61.0%). Programming or debugging was the least common purpose (17.4%), while 19.3% of respondents reported using GenAI for chat or emotional companionship.

### 4.2. Overall Use Frequency and Purpose-Specific Associations

Panel A of Table 3 presents the regression models for depressive symptoms. Overall GenAI use frequency was not associated with depressive symptoms after covariate adjustment in Model 1 (β = −0.016). Adding the eight use-purpose indicators increased R^2^ from 0.038 to 0.059 and revealed clear differences across activities. Information search (β = −0.089), translation (β = −0.058), and creative generation (β = −0.048) were associated with fewer depressive symptoms, whereas writing assistance (β = 0.060), programming or debugging (β = 0.057), and chat or companionship (β = 0.039) were associated with more symptoms. After self-perceived AI competence, literacy, and unavailability anxiety were added in Model 3, the coefficients for information search, translation, writing assistance, and programming remained statistically significant, whereas those for creative generation and chat or companionship were attenuated. The purpose-specific estimates therefore indicate heterogeneity that is not captured by overall use frequency; the coefficients for information search and translation did not differ significantly in a formal contrast test (*p* = 0.263).

The engagement-quality indicators showed stronger and more consistent associations than overall frequency. Higher self-perceived AI competence (β = −0.084) and AI literacy (β = −0.100) were each associated with fewer depressive symptoms, whereas AI unavailability anxiety was associated with more symptoms (β = 0.235). Model 3 explained 12.8% of the variance in CES-D-10 scores.

Panel B of Table 3 shows the corresponding models for life satisfaction. The contrast between overall frequency and engagement quality was similar, although the use-purpose pattern was less differentiated. Overall frequency was not associated with life satisfaction in Model 1 (β = −0.030). Translation was positively associated with life satisfaction, whereas writing assistance and chat or companionship were negatively associated. In Model 3, self-perceived AI competence (β = 0.110) and AI literacy (β = 0.124) were positively associated with life satisfaction, while AI unavailability anxiety was negatively associated (β = −0.067). The frequency coefficient became more negative after these variables were entered (β = −0.045, compared with β = −0.031 in Model 2). Holding the use-purpose indicators and covariates constant, the corresponding standardized coefficient difference was −0.014 (95% bootstrap CI [−0.026, −0.003]), consistent with a suppression-like pattern involving indirect associations in the opposite direction.

### 4.3. Competing Statistical Indirect Pathways

The associations between use frequency and the three intermediate measures were consistent with H1. More frequent GenAI use was associated with higher self-perceived AI competence (β = 0.204), higher AI literacy (β = 0.069), and greater AI unavailability anxiety (β = 0.151). In the simultaneous outcome models, perceived competence and literacy were associated with more favorable psychological well-being, whereas unavailability anxiety was associated with poorer well-being, in line with H2.

Table 4 reports the bootstrapped indirect associations. For depressive symptoms, the pathway through self-perceived AI competence was negative (β = −0.017, 95% CI [−0.025, −0.009]) and the pathway through AI literacy was also negative (β = −0.008, 95% CI [−0.013, −0.003]). By contrast, the pathway through AI unavailability anxiety was positive (β = 0.037, 95% CI [0.027, 0.048]). All three specific confidence intervals excluded zero, indicating competing directions of association, but the total indirect association was not significant (β = 0.013, 95% CI [−0.001, 0.027]).

Life satisfaction showed the same three pathways in the opposite outcome direction. The indirect association through self-perceived AI competence was positive (β = 0.022, 95% CI [0.014, 0.032]), as was the association through AI literacy (β = 0.008, 95% CI [0.004, 0.014]), whereas the association through AI unavailability anxiety was negative (β = −0.011, 95% CI [−0.017, −0.005]). Each specific bootstrap interval excluded zero. In contrast to the depressive-symptom model, the total indirect association was positive (β = 0.020, 95% CI [0.008, 0.032]).

### 4.4. The Moderating Role of Psychological Capital

**Figure 2:**
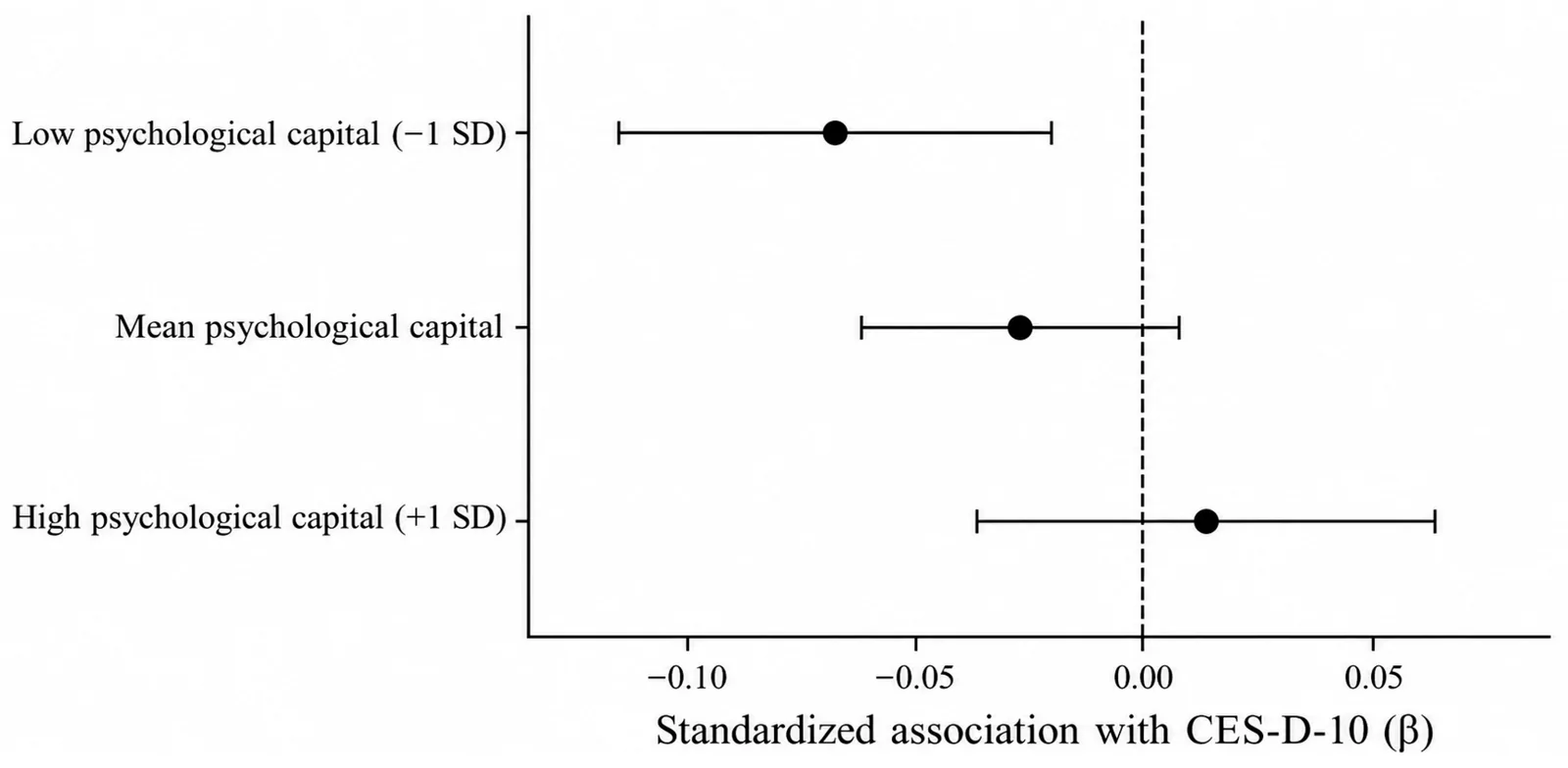
Standardized simple slopes (β) for the association between self-perceived AI competence and depressive symptoms at low, mean, and high psychological capital; error bars show 95% confidence intervals.

H4 predicted that the inverse association involving perceived competence would be stronger among students with lower psychological capital. The interaction between self-perceived AI competence and psychological capital was statistically significant (standardized interaction β = 0.041, *p* = 0.020). Because the main association between perceived competence and depressive symptoms was negative, the positive interaction indicates that the inverse association weakened as psychological capital increased. At −1 SD of psychological capital, perceived competence was associated with fewer depressive symptoms (standardized simple slope β = −0.068); the slopes at the mean (β = −0.027) and +1 SD (β = 0.014) were not statistically significant. Figure 2 illustrates this pattern. The nonsignificant simple slopes at the mean and high psychological capital levels do not establish that their associations are zero.

The standardized index of moderated mediation was β = 0.008, with a 95% bootstrap CI of [0.001, 0.016]. The standardized conditional indirect association from GenAI use frequency to depressive symptoms through perceived competence was β = −0.014 at low psychological capital, and its bootstrap interval excluded zero. The corresponding estimates at the mean (β = −0.005) and high psychological capital (β = 0.003) were not significant. Taken together, the findings are consistent with the resource-substitution pattern anticipated in H4.

### 4.5. Family Resources and Digital Inequality

Table 5 presents the family-resource models. Economic resources were positively associated with self-perceived AI competence (β = 0.102, q < 0.001), whereas cultural resources were positively associated with AI literacy (β = 0.112, q < 0.001). Network resources were positively associated with both perceived competence (β = 0.082, q < 0.001) and AI literacy (β = 0.072, q = 0.002). These associations were consistent with H5. Among the exploratory estimates, economic resources were also positively associated with use frequency (β = 0.068, q = 0.007). None of the resource composites showed a robust association with AI unavailability anxiety after multiple-testing correction.

The resource pattern was therefore more consistent for the quality of engagement than for the reliance-related risk indicator. Economic resources were most clearly related to self-perceived AI competence, cultural resources to critical literacy, and network resources to both. The findings point to differentiated rather than uniform socioeconomic gradients in GenAI engagement. In the unadjusted quartile contrasts (Table S3), self-perceived AI competence averaged 3.415 in the bottom and 3.691 in the top economic-resource quartile, while AI literacy averaged 3.186 and 3.449 in the bottom and top cultural-resource quartiles, respectively. These contrasts describe differences across resource strata rather than a discontinuous threshold. An additional parental-education comparison found modest competence and literacy differences between students with and without a parent holding a bachelor’s degree (Table S7); these descriptive contrasts do not identify a sharp threshold. Because the single-item network-resource indicator has many tied values, the nominal quartile cutoffs split this variable into two much larger strata; these are not strict bottom and top 25% groups (Table S3).

Agricultural-hukou students reported slightly lower mean self-perceived AI competence than non-agricultural-hukou students (3.528 vs. 3.612, *p* = 0.003), as well as lower AI literacy (3.298 vs. 3.391, *p* < 0.001). However, the hukou main effects attenuated after adjustment for family resources and institutional characteristics, and none of the six adjusted hukou-by-resource interaction terms was statistically significant (all *p* > 0.30; Table S5). Full unadjusted hukou contrasts, including group sizes and confidence intervals, are reported in Table S4.

### 4.6. Sensitivity Analyses

The main findings were broadly similar under stricter specifications. Adding academic-major fixed effects yielded standardized indirect associations of β = −0.018 through perceived competence, β = −0.007 through literacy, and β = 0.037 through unavailability anxiety; the standardized competence × psychological capital interaction remained significant (β = 0.043, *p* = 0.015). Restricting the analysis to first-year students also produced a positive and statistically significant interaction (β = 0.043, *p* = 0.022). Alternative measures of AI literacy and AI unavailability anxiety gave substantively similar directions in the principal models. In a residual-correlation sensitivity analysis allowing hypothetical unmeasured mediator–outcome confounding, the 95% bootstrap intervals for the depressive-symptom indirect associations through perceived competence and literacy first included zero at ρ = −0.044 and ρ = −0.068, respectively, compared with ρ = 0.213 for unavailability anxiety. For life satisfaction, the corresponding critical values were ρ = 0.066, 0.076, and −0.038. These scenarios showed greater sensitivity of the perceived competence and literacy paths to the specified residual correlations (Table S6). The sensitivity analysis does not rule out unmeasured confounding or establish a temporal indirect effect.

## 5. Discussion

### 5.1. Main Findings and the Quality of GenAI Engagement

Drawing on data from 3283 university students predominantly enrolled in Guangxi, this study examined GenAI engagement by distinguishing overall frequency from use purpose and engagement quality. The findings indicate that frequency alone provides an incomplete account of students’ psychological adjustment. Overall use frequency was not associated with depressive symptoms or life satisfaction in the baseline models, whereas several specific uses showed distinct associations. More frequent use was also related to greater self-perceived AI competence and literacy, but at the same time to greater anxiety when AI was unavailable. These dimensions were associated with well-being in opposite directions. In addition, the perceived competence–depression association was strongest among students with lower psychological capital, and family resources were differentially associated with competence and literacy.

The contrast between overall frequency and purpose-specific use is consistent with the broader move in digital-well-being research away from simple exposure measures. Orben and Przybylski (2019) show that aggregate technology-use measures can produce small average associations even when experiences differ substantially across users. The differential-susceptibility model offers a related explanation by locating media effects within characteristics of the user and the context of exposure (Valkenburg & Peter, 2013). On this view, the absence of a baseline frequency association does not imply that GenAI is unrelated to well-being. It suggests instead that a single frequency score combines activities with different functions and meanings. This interpretation is compatible with dynamic accounts of digital well-being (Vanden Abeele, 2021) and with evidence that the consequences of digital engagement vary across forms of use (Valkenburg, 2022). The present results extend this reasoning to GenAI: information search, translation, writing assistance, programming, and companionship were not psychologically interchangeable activities.

The results for perceived AI competence and literacy further reinforce the distinction between exposure and capability. Long and Magerko (2020) conceptualize AI literacy as a capacity to understand and critically engage with AI rather than simply to access it. Ng et al. (2021) similarly place evaluation and ethical awareness alongside application skills. Although the present three-item index captures only part of this broader construct, higher literacy was consistently associated with fewer depressive symptoms and greater life satisfaction. This pattern accords with recent work linking AI literacy to adaptive learning and well-being in GenAI-supported higher education (Shi et al., 2025). Multinational evidence also shows substantial variation in AI literacy among university students despite widespread access to AI tools (Hornberger et al., 2025). These findings suggest that access and frequency are poor substitutes for the self-reported capacities with which students evaluate and manage AI-mediated work.

AI unavailability anxiety showed the opposite pattern. Students who reported greater distress when AI was unavailable also reported more depressive symptoms and lower life satisfaction. This result is consistent with work that distinguishes anxiety about AI from acceptance or competence (Cengiz & Peker, 2025). Research on problematic AI use under academic stress points to a related form of reliance (S. Zhang et al., 2024), while Tian and Zhang (2025) draw attention to dependence-related costs for critical thinking. Frequent use may coexist with anxiety concerning uninterrupted AI access, a response distinct from competence or literacy. Work on cognitive offloading points to a similar tension between the convenience of delegation and concern about reduced cognitive ownership (Su et al., 2026). The competing indirect pathways therefore provide one plausible explanation for the weak overall frequency association: capability gains and reliance-related discomfort may operate in opposite directions.

### 5.2. Psychological Resources and Digital Inequality

The moderation results add a further layer to this interpretation. Self-perceived AI competence was most strongly associated with fewer depressive symptoms among students with lower psychological capital. Resource-substitution theory proposes that the marginal value of one resource may increase when another is scarce (Ross & Mirowsky, 2006). Students with greater confidence, optimism, resilience, and goal-directedness may already have resources for managing uncertainty and difficult academic tasks. For students with fewer such resources, perceived AI competence may coincide with an additional sense of control. The interaction remained evident after adjustment for academic major and within the first-year subsample.

Family resources were also associated with the quality of GenAI engagement, although the pattern differed across resource dimensions. Economic and network resources were related to higher self-perceived AI competence, while cultural and network resources were related to higher AI literacy. These differences are consistent with the development of digital-divide theory from unequal access toward unequal skills and outcomes. Hargittai (2002) drew early attention to skill-based inequalities, and van Deursen and Helsper (2015) later emphasized differences in the benefits users obtain from digital technologies. Helsper (2021) situates both forms of inequality within broader distributions of social resources. The present findings suggest that this framework remains relevant when access to GenAI itself is becoming commonplace. Hukou groups also differed descriptively in competence and literacy, while adjusted hukou-by-resource interactions were not statistically significant.

Material resources may make devices, subscriptions, and repeated practice easier to obtain; cultural resources may support evaluative habits; and network resources may connect students with more experienced users. Tang (2025) similarly identifies social and cultural inequalities in college students’ GenAI performance. C. Zhang et al. (2025) show that the perceived educational benefits of ChatGPT also vary across student groups. In the present study, self-perceived AI competence and self-reported AI literacy varied across family-resource backgrounds and were associated with psychological well-being. Expanding access to GenAI may therefore be insufficient if differences in the capacity to use and evaluate it remain unaddressed.

### 5.3. Theoretical and Practical Implications

Theoretically, the findings support a shift from an exposure-centered account of GenAI toward an engagement-quality perspective. Treating frequency, purpose, perceived competence, literacy, and unavailability anxiety as distinct dimensions helps explain why the same level of use may be associated with different psychological well-being outcomes. The parallel-path results further show that competence-related resources and reliance-related discomfort can coexist rather than form opposite ends of a single continuum. The moderation and family-resource findings extend this perspective by locating engagement quality within students’ existing psychological and social resources. In this sense, GenAI use is not simply an individual behavioral choice; its associations with well-being depend on the competencies students bring to the technology, the forms of reliance that accompany use, and the resources available to support high-quality engagement.

From a practical perspective, universities should treat AI literacy as more than prompting proficiency. Instruction should develop students’ ability to evaluate evidence, uncertainty, bias, and privacy risk, as well as their judgment about when work should remain independent or involve human consultation. UNESCO (2023) similarly emphasizes human agency and critical oversight in educational uses of GenAI. Assignments requiring students to verify AI outputs, explain their reasoning, and identify uncertainty can integrate critical evaluation into regular coursework. At the institutional level, universities could establish centrally managed AI access arrangements, including campus-wide licensing or shared platforms with clear privacy and academic-integrity standards, to reduce differences in access to suitable tools.

Universities should also preserve students’ ability to work and regulate emotion when AI is unavailable, unreliable, or inappropriate. Courses can combine AI-assisted and unassisted tasks and ask students to document how AI contributed to a solution, encouraging flexible use rather than routine delegation. For students without ready access to experienced AI users, universities could embed structured peer mentoring, trained teaching-assistant support, and dedicated AI consultation desks within required courses. Coordination among teaching units, libraries, and student-support services would make this assistance a routine part of the educational environment. Such arrangements could give particular attention to students facing both limited family resources and lower psychological capital, whose opportunities to acquire AI-related competence may be especially constrained. First-year courses could include confidential, opt-in needs screening and proactively offer peer assistance to students without established support networks, rather than relying on self-referral alone. Offering foundational AI-learning support to all students within required courses, while making individualized follow-up voluntary and confidential, may reduce reliance on self-identification and avoid stigmatizing those with fewer social connections.

### 5.4. Limitations and Future Research

Several limitations should be considered. First, the cross-sectional design cannot establish temporal ordering or exclude reverse causation, and the indirect associations were sensitive to hypothetical unmeasured mediator–outcome confounding. Second, the measures were primarily self-reported. The single-item indicators of self-perceived AI competence, AI unavailability anxiety, and global life satisfaction do not permit internal-consistency reliability estimation. Although published work provides validity evidence for single-item global life satisfaction, the competence and unavailability-anxiety indicators cover narrower portions of their respective constructs and were not independently validated in the present sample. The size and direction of any resulting measurement-error bias cannot be determined from these data, and perceived competence may differ from objective performance. The three-item AI-literacy measure likewise captures selected aspects of a broader construct. Third, although the Chinese PPQ has an established four-dimensional basis and showed acceptable model fit in this sample, its five-point response format differs from the original instrument and structural-fit results do not by themselves establish equivalence across response formats. Finally, the survey included students from several universities in Guangxi; future research should broaden recruitment to other regions of China to examine the generalizability of the findings. Parental-education contrasts are descriptive, and the survey does not separately identify specific household digital devices. The measurement rationale and the limits of construct-validity evidence for these single-item indicators are explained further in Supplementary Methods S1.7.

These limitations also indicate useful directions for further research. Longitudinal and experience-sampling designs could establish whether changes in GenAI use precede changes in competence, unavailability anxiety, and well-being and could distinguish brief instrumental use from more sustained reliance. Combining self-report measures with objective performance tasks and behavioral logs would clarify whether perceived competence corresponds to effective and critical use. Randomized training studies could test whether AI-literacy instruction improves both task performance and psychological adaptation and whether the benefits differ by psychological capital or family resources. Multi-institutional research across different regions of China could examine whether the present patterns vary by local educational conditions and family-resource environments. Person-centered analyses may also be useful in future datasets with richer measures of use intensity, dependence, and task context, where distinct configurations of competence, literacy, and anxiety can be estimated more reliably. Future multiwave studies should combine validated multi-item AI measures with objective prompt-design and error-detection tasks and recruit universities in economically diverse cities, including Beijing and Shanghai, to assess temporal ordering and possible regional or hukou differences.

## 6. Conclusions

This study suggests that the relationship between GenAI engagement and university students’ psychological well-being is better understood through engagement quality than through frequency alone. Overall use frequency showed little association with depressive symptoms or life satisfaction in baseline models, whereas specific use purposes and engagement-quality indicators differentiated outcomes. Higher self-perceived AI competence and self-reported AI literacy were associated with more favorable psychological well-being, while AI unavailability anxiety was associated with poorer well-being. Their opposing statistical indirect pathways partly offset one another for depressive symptoms, with a nonsignificant total indirect association. The inverse association between perceived competence and depressive symptoms was more pronounced at lower levels of psychological capital, consistent with a resource-substitution interpretation. Family economic, cultural, and network resources were differentially associated with self-perceived competence and AI literacy, alongside modest descriptive hukou differences. Together, these findings shift attention beyond the frequency of GenAI use toward institutional support for critical, autonomous, and equitable engagement, including accessible AI tools, course-embedded literacy instruction, and structured peer assistance.

## Figures and Tables

**Table 1:** Descriptive statistics and internal consistency of focal variables.

Variable	*N*	*M*	*SD*	Cronbach’s α
AI use frequency	3283	4.268	0.792	—
Self-perceived AI competence	3283	3.553	0.725	—
AI literacy	3283	3.326	0.545	0.826
AI unavailability anxiety	3283	2.854	0.764	—
CES-D-10 depressive symptoms	3283	8.871	4.974	0.838
Life satisfaction	3283	3.756	0.761	—
Psychological capital	3283	3.243	0.464	0.927
Economic resources (z)	3283	0.000	1.000	—
Cultural resources (z)	3283	0.000	1.000	—
Network resources (z)	3283	0.000	1.000	—

Note. Internal-consistency coefficients are reported only for multi-item indices. AI literacy uses three self-reported items; CES-D-10 positive-affect items were reverse-scored before summation (range 0–30); psychological capital was measured with the 26-item Chinese PPQ (K. Zhang et al., 2010).

**Table 2:** Prevalence of generative AI use purposes.

AI Use Purpose	*N*	%
Information search	2855	87.0
Translation	2002	61.0
Writing assistance	2153	65.6
Programming/debugging	570	17.4
Data analysis	1249	38.0
Creative generation	1457	44.4
Long-document/video summarization	916	27.9
Chat/emotional companionship	635	19.3

Note. Use-purpose indicators were multiple-response binary items; percentages therefore do not sum to 100%.

**Table 3:** Generative AI engagement and psychological well-being.

Variable	Model 1	Model 2	Model 3
**Panel A. Depressive symptoms (CES-D-10)**			
AI use frequency	−0.016 (0.020)	0.002 (0.020)	−0.016 (0.020)
Information search	—	−0.089 *** (0.018)	−0.059 ** (0.018)
Translation	—	−0.058 ** (0.018)	−0.049 ** (0.017)
Writing assistance	—	0.060 ** (0.018)	0.049 ** (0.018)
Programming/debugging	—	0.057 ** (0.021)	0.045 * (0.020)
Data analysis	—	−0.033 (0.019)	−0.028 (0.018)
Creative generation	—	−0.048 ** (0.018)	−0.033 (0.018)
Long-document summarization	—	−0.029 (0.020)	−0.021 (0.019)
Chat/companionship	—	0.039 * (0.019)	0.030 (0.018)
Self-perceived AI competence	—	—	−0.084 *** (0.019)
AI literacy	—	—	−0.100 *** (0.020)
AI unavailability anxiety	—	—	0.235 *** (0.019)
Controls	Yes	Yes	Yes
Grade fixed effects	Yes	Yes	Yes
Institution fixed effects	Yes	Yes	Yes
N	3283	3283	3283
R^2^	0.038	0.059	0.128
**Panel B. Life satisfaction**			
AI use frequency	−0.030 (0.020)	−0.031 (0.020)	−0.045 * (0.021)
Information search	—	0.016 (0.017)	−0.006 (0.018)
Translation	—	0.055 ** (0.017)	0.050 ** (0.017)
Writing assistance	—	−0.056 ** (0.018)	−0.058 ** (0.018)
Programming/debugging	—	0.030 (0.020)	0.036 (0.020)
Data analysis	—	0.022 (0.019)	0.016 (0.019)
Creative generation	—	0.030 (0.018)	0.019 (0.018)
Long-document summarization	—	−0.001 (0.019)	−0.010 (0.019)
Chat/companionship	—	−0.038 * (0.018)	−0.036 * (0.018)
Self-perceived AI competence	—	—	0.110 *** (0.020)
AI literacy	—	—	0.124 *** (0.021)
AI unavailability anxiety	—	—	−0.067 *** (0.018)
Controls	Yes	Yes	Yes
Grade fixed effects	Yes	Yes	Yes
Institution fixed effects	Yes	Yes	Yes
N	3283	3283	3283
R^2^	0.054	0.063	0.098

Note. HC3 robust standard errors are given in parentheses. All models adjust for the stated covariates, grade and institution fixed effects. * *p* < 0.05, ** *p* < 0.01, *** *p* < 0.001.

**Table 4:** Covariate-adjusted parallel indirect associations.

Outcome	Indirect Path	β	Bootstrap 95% CI
Depressive symptoms	AI frequency → self-perceived AI competence → outcome	−0.017 *	[−0.025, −0.009]
Depressive symptoms	AI frequency → AI literacy → outcome	−0.008 *	[−0.013, −0.003]
Depressive symptoms	AI frequency → AI unavailability anxiety → outcome	0.037 *	[0.027, 0.048]
Depressive symptoms	Total indirect association	0.013	[−0.001, 0.027]
Life satisfaction	AI frequency → self-perceived AI competence → outcome	0.022 *	[0.014, 0.032]
Life satisfaction	AI frequency → AI literacy → outcome	0.008 *	[0.004, 0.014]
Life satisfaction	AI frequency → AI unavailability anxiety → outcome	−0.011 *	[−0.017, −0.005]
Life satisfaction	Total indirect association	0.020 *	[0.008, 0.032]

Note. Percentile 95% confidence intervals are based on 5000 bootstrap resamples, with the three intermediate variables estimated simultaneously. * indicates a 95% bootstrap confidence interval that excludes zero.

**Table 5:** Family resource inequalities in GenAI engagement quality.

Outcome	Resource Predictor	β (SE)	BH-FDR q	R^2^
AI use frequency	Economic resources	0.068 ** (0.023)	0.007	0.063
AI use frequency	Cultural resources	−0.053 * (0.025)	0.074	0.063
AI use frequency	Network resources	0.037 (0.020)	0.101	0.063
Self-perceived AI competence	Economic resources	0.102 *** (0.023)	<0.001	0.040
Self-perceived AI competence	Cultural resources	0.040 (0.025)	0.123	0.040
Self-perceived AI competence	Network resources	0.082 *** (0.021)	<0.001	0.040
AI literacy	Economic resources	0.029 (0.022)	0.200	0.056
AI literacy	Cultural resources	0.112 *** (0.025)	<0.001	0.056
AI literacy	Network resources	0.072 *** (0.021)	0.002	0.056
AI unavailability anxiety	Economic resources	−0.009 (0.022)	0.701	0.015
AI unavailability anxiety	Cultural resources	−0.045 (0.025)	0.101	0.015
AI unavailability anxiety	Network resources	0.036 (0.021)	0.118	0.015

Note. Economic, cultural, and network resources were entered simultaneously. Models adjust for gender, hukou, grade fixed effects, and institution fixed effects. HC3 robust standard errors are given in parentheses. BH-FDR q values account for 12 resource comparisons; stars refer to unadjusted *p* values: * *p* < 0.05, ** *p* < 0.01, *** *p* < 0.001.

## Data Availability

The data supporting the findings of this study are available from the corresponding author upon reasonable request, subject to applicable ethical and privacy restrictions.

## Supplementary Materials

The following supporting information can be downloaded at: https://www.mdpi.com/article/10.3390/bs16101942/s1, Table S1: Ordinal CFA model fit for two Chinese PPQ specifications; Table S2: Internal consistency of the Chinese 26-item PPQ; Table S3: Lower and upper family-resource quartiles: unadjusted differences; Table S4: Unadjusted differences by household registration; Table S5: Hukou × family-resource interactions; Table S6: Indirect-path estimates and residual-correlation sensitivity; Table S7: Parental bachelor’s education: descriptive differences in GenAI engagement. Supplementary Methods S1 (Statistical model specification and calculations) contains S1.1: Regression and standardization; S1.2: Parallel indirect-path models; S1.3: Moderated mediation and Hayes index; S1.4: Hypothetical residual-correlation sensitivity; S1.5: Ordinal PPQ factor models; S1.6: Interpretable resource-group comparison; S1.7: Rationale and limitations of single-item indicators.
