# Supplementary material for: Beyond Frequency: Generative AI Engagement Quality, Psychological Well-Being, and Digital Inequality Among Chinese University Students
Source: Behav Sci (Basel). 2026 Oct 19;16(10):1942. doi: 10.3390/bs16101942 (PMC13701433; doi:10.3390/bs16101942)
Supplement: Supplementary file 1 [file behavsci-16-01942-s001.zip › behavsci-4616224-supplementary.pdf]

Supplementary Materials

Table S1. Ordinal CFA model fit for two Chinese PPQ specifications

PPQ model	df	CFI	TLI	RMSEA	RMSR
Correlated four-factor + wording factor	288	0.994	0.994	0.057	0.050
Second-order + wording factor	290	0.992	0.991	0.066	0.056

Note. N = 3,283. Both models were estimated using polychoric correlations and the same diagonally weighted least squares (DWLS) procedure, with an orthogonal wording-method factor for items 8, 10, 12, 14, and 25. RMSR is the root mean square residual for the polychoric correlations. The unscaled DWLS chi-square statistic is sensitive to sample size and is not treated here as a robust chi-square test. Comparative fit indices were computed against a common independence baseline.

Table S2. Internal consistency of the Chinese 26-item PPQ

Dimension	No. items	Cronbach's α
Self-efficacy	7	0.898
Resilience	7	0.719
Hope	6	0.838
Optimism	6	0.890
Full PPQ	26	0.927

Note. Items were scored on five response categories. Reverse-keyed items: 8, 10, 12, 14, and 25. Resilience (α = 0.719) shows acceptable, although lower, internal consistency relative to the other PPQ dimensions.

Table S3. Lower and upper family-resource quartiles: unadjusted differences

Resource	Outcome	N low	N high	M low	M high	ΔM	95% CI for ΔM	p
Economic	Self-perceived AI competence	1080	883	3.415	3.691	0.276	[0.210, 0.342]	<0.001
Economic	AI literacy	1080	883	3.252	3.439	0.186	[0.137, 0.236]	<0.001
Cultural	Self-perceived AI competence	822	832	3.440	3.669	0.229	[0.157, 0.301]	<0.001
Cultural	AI literacy	822	832	3.186	3.449	0.263	[0.210, 0.316]	<0.001
Network	Self-perceived AI competence	1655	1628	3.466	3.643	0.177	[0.127, 0.226]	<0.001
Network	AI literacy	1655	1628	3.269	3.383	0.114	[0.077, 0.152]	<0.001

Note. Upper and lower groups include observations tied at the quartile boundaries. ΔM = upper-group mean minus lower-group mean; 95% CIs and p values are from two-sided Welch tests. Quartile comparisons focus on self-perceived competence and AI literacy, the outcomes in H5; the corresponding adjusted associations for unavailability anxiety were not robust after multiple-testing correction (main-text Table 5). In particular, the standardized network-resource variable derives from a discrete single-item response: ties at the nominal lower and upper quartile boundaries produce groups of 1,655 and 1,628 (together N = 3,283). Thus, these network-resource strata are not literal bottom and top quartiles, and the contrasts should not be interpreted as estimates of an identified threshold.

Table S4. Unadjusted differences by household registration

Outcome	N agri.	N non-agri.	M agri.	M non-agri.	ΔM	95% CI	p
Self-perceived AI competence	2296	987	3.528	3.612	0.084	[0.028, 0.139]	0.003
AI literacy	2296	987	3.298	3.391	0.093	[0.052, 0.135]	<0.001

Note. ΔM = non-agricultural mean minus agricultural mean; 95% CIs and p values are based on two-sided Welch tests.

Table S5. Hukou × family-resource interactions

Outcome	Interaction	β	95% CI	p	BH-FDR q
Self-perceived AI competence	Economic × hukou	−0.017	[−0.077, 0.043]	0.576	0.691
Self-perceived AI competence	Cultural × hukou	−0.029	[−0.097, 0.039]	0.397	0.691
Self-perceived AI competence	Network × hukou	0.027	[−0.025, 0.080]	0.304	0.691
AI literacy	Economic × hukou	−0.001	[−0.057, 0.055]	0.971	0.971
AI literacy	Cultural × hukou	−0.036	[−0.106, 0.034]	0.311	0.691
AI literacy	Network × hukou	0.019	[−0.034, 0.072]	0.478	0.691

Note. Hukou: 0 = agricultural, 1 = non-agricultural. β and its 95% CI are standardized for the resource × hukou product. Resource dimensions and interactions were entered jointly, adjusting for gender, grade and institution fixed effects; HC3 robust inference. BH-FDR q values cover six interaction tests.

Table S6. Indirect-path estimates and residual-correlation sensitivity

Outcome	Statistical path via	β	Bootstrap 95% CI	Critical ρ (rho)
Depressive symptoms	Self-perceived AI competence	−0.017	[−0.025, −0.009]	−0.044
Depressive symptoms	AI literacy	−0.008	[−0.013, −0.003]	−0.068
Depressive symptoms	AI unavailability anxiety	0.037	[0.027, 0.048]	0.213
Life satisfaction	Self-perceived AI competence	0.022	[0.014, 0.032]	0.066
Life satisfaction	AI literacy	0.008	[0.004, 0.014]	0.076
Life satisfaction	AI unavailability anxiety	−0.011	[−0.017, −0.005]	−0.038
Depressive symptoms	Total indirect association	0.013	[−0.001, 0.027]	—
Life satisfaction	Total indirect association	0.020	[0.008, 0.032]	—

Note. β and percentile bootstrap 95% CIs are fully standardized (5,000 resamples). Critical ρ (rho) is the signed hypothetical mediator–outcome residual correlation at which a specific path's 95% bootstrap CI first includes zero; positive and negative signs indicate the direction of that correlation, not an effect direction. Other mediator–outcome residual correlations were fixed at zero. Critical ρ (rho) is not defined for the total indirect association.

Table S7. Parental bachelor's education: descriptive differences in GenAI engagement

Outcome	Parent BA+ n = 427	Neither parent BA+ n = 2,856	ΔM	95% CI	p
Self-perceived AI competence	3.642	3.540	0.101	[0.021, 0.182]	0.014
AI literacy	3.419	3.312	0.108	[0.046, 0.169]	<0.001

Note. BA+ means at least one parent completed a bachelor's degree or higher (category 5 or 6 of the six-category parental-education item). Two-sided Welch mean tests and 95% confidence intervals are unadjusted. Group contrasts do not test for a causal or nonlinear threshold.

Supplementary Methods S1. Statistical model specification and calculations

S1.1. Regression and standardization

For each outcome (depressive symptoms and life satisfaction, estimated separately), Model 1 includes GenAI frequency F and covariates C; Model 2 adds eight use-purpose indicators; Model 3 additionally includes the three engagement indicators. HC3 robust standard errors are computed before rescaling. For any predictor X_j with unstandardized estimate b_j, the fully standardized coefficient is:

$$\beta_j^{\mathrm{std}} = b_j \frac{\mathrm{SD}(X_j)}{\mathrm{SD}(Y)}$$

S1.2. Parallel indirect-path models

The main parallel-path equations are given in Section 3.3. For mediator k, a is the frequency-to-mediator coefficient and b is its coefficient in the outcome regression. The specific and total statistical indirect associations are:

$$I_k = a_k b_k, \qquad I_{\mathrm{total}} = \sum_{k=1}^{3} a_k b_k \quad I_k = a_k b_k, \qquad I_{\mathrm{total}} = \sum_{k=1}^{3} a_k b_k$$

$$I_k^{\mathrm{std}} = a_k b_k \frac{\mathrm{SD}(F)}{\mathrm{SD}(Y)}$$

All 95% confidence intervals are percentile intervals from 5,000 nonparametric bootstrap samples, re-estimating the component regressions and full standardization in every draw. Because the dataset is cross-sectional, the terms refer to statistical indirect associations.

S1.3. Moderated mediation and Hayes index

In the depressive-symptom model, W represents mean-centered psychological capital, and M1 represents self-perceived AI competence. The moderated outcome regression, conditional indirect association, and Hayes index are: This competence-path representation is reported separately from the three-mediator parallel indirect-path model in Section 3.3; it does not specify moderation of the literacy and unavailability-anxiety pathways.

$$Y_i = \beta_0 + c' F_i + b_1 M_{1i} + dW_i + b_{1,\mathrm{PC}}(M_{1i}W_i) + \boldsymbol{\gamma}^\mathsf{T}\mathbf{C}_i + \varepsilon_i$$

$$I_1(W) = a_1\big(b_1 + b_{1,\mathrm{PC}}W\big), \qquad \mathrm{Index}_{\mathrm{MM}} = a_1 b_{1,\mathrm{PC}}$$

$$\mathrm{Index}_{\mathrm{MM}}^{\mathrm{std}} = a_1 b_{1,\mathrm{PC}}\,\frac{\mathrm{SD}(F)\,\mathrm{SD}(W)}{\mathrm{SD}(Y)}$$

The interaction coefficient in these expressions is the competence × psychological-capital term predicting depressive symptoms. Conditional simple slopes are evaluated at low (−1 SD), mean, and high (+1 SD) psychological capital. The final equation standardizes the Hayes index using the standard deviations of use frequency, psychological capital, and depressive symptoms, with all paths re-estimated over 5,000 bootstrap samples.

Derivation of the index: the conditional competence-path association is $I_1(W) = a_1(b_1 + b_1$,PCW). Under the specified model, a_1 does not vary with W. Differentiating the conditional product with respect to W gives $\partial I_1(W)/\partial W = a_1b_1$,PC (Hayes's index). The fully standardized index is this derivative multiplied by SD(F) × SD(W) / SD(Y); its confidence interval is obtained by applying the same calculation in each of the 5,000 bootstrap samples.

S1.4. Hypothetical residual-correlation sensitivity

The sensitivity parameter represents the hypothetical correlation between the disturbance of a specified mediator equation and the outcome-model disturbance:

$$\mathrm{Cov}(u_k, \varepsilon) = \rho_k\,\sigma_{u_k}\sigma_\varepsilon$$

Let capital omega denote the 3 × 3 covariance matrix of the mediator residuals, and let the vector nu contain the mediator–outcome disturbance covariances, with only the entry for the varied mediator potentially nonzero. The corresponding linear-projection adjustment is:

Algebraic clarification: after residualizing the mediator and outcome equations with respect to frequency and covariates, the fitted mediator coefficient vector under the reference zero-correlation scenario can be written as $b(0) = b^* + \Omega^{-1}\nu$, where b^* denotes the slope vector under a posited mediator–outcome disturbance covariance ν. Rearranging gives $b(\rho k) = b(0) - \Omega^{-1}\nu k$. For mediator k, νk has kth component $\rho k\sigma_u k\sigma_e$ and zeros elsewhere. This is a linear-projection sensitivity adjustment, not an identification proof.

$$\mathbf{b}(\rho_k) = \mathbf{b}(0) - \boldsymbol{\Omega}^{-1}\boldsymbol{\nu}_k$$

The other two mediator–outcome residual correlations remain fixed at zero in each scenario. The reported critical correlation is the first tested value at which the 95% bootstrap interval for that indirect association includes zero (Table S6). This sensitivity analysis assesses only hypothetical mediator–outcome residual confounding, not unmeasured confounding of the use-frequency–mediator or use-frequency–outcome relationships, and cannot identify causal mediation. Operationally, the critical-value criterion is evaluated along the posited signed correlation for one mediator at a time, checking when the adjusted specific-indirect-association percentile interval first contains zero. The reported three-decimal critical correlations are numerical approximations to confidence-interval crossing points in these specified one-at-a-time confounding scenarios; they are not theoretical or causal thresholds.

S1.5. Ordinal PPQ factor models

The 26 PPQ five-category items were reverse-keyed as described in Section 3.2.6. Both CFA models used 325 pairwise polychoric correlations, diagonal inverse-variance weights, and a factor orthogonal to the four substantive factors for the five reverse-keyed items. The model-implied latent-response correlation matrix and DWLS discrepancy are:

$$\mathbf{\Sigma} = \mathbf{\Lambda}\mathbf{\Phi}\mathbf{\Lambda}^{\mathsf{T}} + \mathbf{\Theta}$$

$$F_{\mathrm{DWLS}} = (\mathbf{r} - \widehat{\mathbf{r}})^{\mathsf{T}}\mathbf{W}_D(\mathbf{r} - \widehat{\mathbf{r}})$$

In the correlated four-factor specification, Φ is freely correlated across self-efficacy, resilience, hope, and optimism; the second-order specification constrains Φ through a single higher-order psychological-capital factor. The same estimation and baseline conventions were used for both models (Table S1).

S1.6. Interpretable resource-group comparison

Parental education was grouped into at least one bachelor's-degree parent versus neither parent holding that qualification. Table S7 reports unadjusted differences in the two engagement outcomes with Welch 95% confidence intervals. The original childhood-assets item is composite and does not separately distinguish ownership of a computer or access to home internet. No sharp threshold, change-point, or causal effect is inferred from these descriptive group comparisons. This BA+ comparison provides an interpretable grouping, not an estimated education threshold; the available indicators do not establish a discontinuity at completion of a bachelor's degree.

S1.7. Rationale and limitations of single-item indicators

Self-perceived AI competence and AI unavailability anxiety were measured using narrowly worded, transparently anchored self-report items because the original survey did not administer corresponding multi-item instruments or performance assessments. The competence item refers to perceived effectiveness in formulating prompts and judging output quality; the anxiety item records discomfort specifically when AI access is interrupted. Neither item is treated as an objective proficiency test, a clinical anxiety measure, or a fully validated multidimensional scale. The absence of repeated observations and parallel multi-item measures precludes test–retest reliability, internal-consistency estimation, and convergent-validity testing for these items in the present sample. Their observed associations with well-being do not independently establish construct validity. The single-item global life-satisfaction measure likewise does not provide sample-specific test–retest or convergent-validity evidence; relevant evidence for similar global items from other samples should not be mistaken for validation of this particular survey item.

Methodological research shows that single global evaluations can sometimes be informative for sufficiently circumscribed constructs, but validity evidence is construct-specific. (Cheung & Lucas, 2014) reported external validation of a single-item global life-satisfaction measure. However, such evidence does not supply reliability estimates for the two GenAI-specific indicators used here. Follow-up research should use validated multi-item measures and objective prompt-design and error-detection tasks.

Reference

Cheung, F., & Lucas, R. E. (2014). Assessing the validity of single-item life satisfaction measures: Results from three large samples. Quality of Life Research, 23(10), 2809–2818. https://doi.org/10.1007/s11136-014-0726-4.
